# Herbicide-Intercalated Zinc Layered Hydroxide Nanohybrid for a Dual-Guest Controlled Release Formulation

**DOI:** 10.3390/ijms13067328

**Published:** 2012-06-13

**Authors:** Mohd Zobir Hussein, Nor Shazlirah Shazlyn Abdul Rahman, Siti H. Sarijo, Zulkarnain Zainal

**Affiliations:** 1 Department of Chemistry, Faculty of Science, Universiti Putra Malaysia, Serdang, Selangor 43400, Malaysia; E-Mails: nsshazlyn@gmail.com (N.S.S.A.R.); zulkar@science.upm.edu.my (Z.Z.); 2 Faculty of Applied Science, Universiti Teknologi MARA (UiTM), Shah Alam, Selangor 40450, Malaysia; E-Mail: siti_halimah_404@yahoo.com; 3 Advanced Materials and Nanotechnology Laboratory, Institute of Advanced Technology (ITMA), Universiti Putra Malaysia, Serdang, Selangor 43400, Malaysia

**Keywords:** 4-(2,4-dichlorophenoxy) butyrate, 2-(3-chlorophenoxy) propionate, zinc layered hydroxide, simultaneous release

## Abstract

Herbicides, namely 4-(2,4-dichlorophenoxy) butyrate (DPBA) and 2-(3-chlorophenoxy) propionate (CPPA), were intercalated simultaneously into the interlayers of zinc layered hydroxide (ZLH) by direct reaction of zinc oxide with both anions under aqueous environment to form a new nanohybrid containing both herbicides labeled as ZCDX. Successful intercalation of both anions simultaneously into the interlayer gallery space of ZLH was studied by PXRD, with basal spacing of 28.7 Å and supported by FTIR, TGA/DTG and UV-visible studies. Simultaneous release of both CPPA and DPBA anions into the release media was found to be governed by a pseudo second-order equation. The loading and percentage release of the DPBA is higher than the CPPA anion, which indicates that the DPBA anion was preferentially intercalated into and released from the ZLH interlayer galleries. This work shows that layered single metal hydroxide, particularly ZLH, is a suitable host for the controlled release formulation of two herbicides simultaneously.

## 1. Introduction

Nanomaterials such as two-dimensional (2D) nanosheets have recently gained much attention due to their unique physical and chemical properties [1]. Excellent intercalation properties of 2D layered material offer a new scope for developing hybrid materials at nanoscale dimensions or the so-called nanocomposite. This type of material offers a variety of applications in industries and the environment such as anion-exchanger [2], catalysis, delamination, as well as in medical science, and more [3,4].

Layered double hydroxides (LDHs) and hydroxy double salts (HDSs) have been studied extensively and are recognized for their anion-exchangeable properties [5,6]. LDHs having brucite-type layers of mixed metal hydroxide can be expressed by the general formula [M(II)_1−x_M(III)_x_(OH)_2_](A^−n^)_x/n_·yH_2_O where M(II) and M(III) are the divalent and trivalent metal cations, respectively. A^n−^ is the exchangeable anion and y represents the water content of the interlayer region. HDSs are identical to LDHs, only the metal hydroxide inorganic layers are composed of two divalent metal cations [7]. Layered hydroxide salts (LHS) such as zinc layered hydroxide (ZLH) are similar to the HDS structure whose structure is similar to that of brucite; however, the inorganic layers are composed of only one type of metal cation [8] such as Mg^2+^, Cu^2+^, Zn^2+^ and Ni^2+^ and can be represented by the general formula, M(II)(OH)_2−x_(A^−n^)_x/n_·yH_2_O. In this structure, the OH^−^ anions on the brucite hydroxide layer are substituted by water molecules and counter anions [9–12].

The gallery structure of LDHs is expandable, if the guest anion is larger in size than the interlayer anion present in the LDHs, or the spatial orientation of the guest warrants the expansion. Various guest anions can be intercalated into the LDH’s gallery structure, thus a variety of hybrid nano-layered materials can be designed [2,13]. Identical to the LDHs phase, ZLH too can undergo anion-exchange reaction, by substituting the negatively charged organic molecules for the exchangeable interlayer anions in the ZLH lattice to form layered nanohybrids [14,15]. Development of ZLH hybrid materials has shown great potential application in industry such as anticorrosion agent and dye-sensitized solar cells [16,17].

To our knowledge, unlike LDHs, the use of ZLH as matrices [15,18,19], particularly in controlled release (CR) formulations, has rarely been studied. In the agricultural sector, the excess amount of pesticide runoff into the surface and groundwater has led to water pollution [20]. Therefore, the CR formulation can be applied to lower the risks of environmental pollution by reducing the amount of pesticides used for the same activity, thus decreasing the nontarget effects [21]. Additionally, CR formulation is superior to its counterpart and results in a higher yield and better crop quality. Such a formulation also finds use in active agents such as drugs [22,23], vitamins [24,25], herbicides, pesticides and plant growth regulators [26,27] in which the active agents are successfully intercalated into layered materials to produce controlled release formulations.

This paper aimed at the formation of phase-pure, well-ordered ZLH-intercalated nanohybrids by the intercalation of phenoxy herbicides-type active agents, namely 4-(2,4-dichlorophenoxy) butyrate (DPBA) and 2-(3-chlorophenoxy) propionate (CPPA) simultaneously into the ZLH interlayer using a simple direct reaction of zinc oxide (ZnO) with the guest anions in an aqueous environment. In our previous study, we reported the successful intercalation of both herbicides anions into LDHs by an anion-exchange method. Our results showed that the loading and release percentages of DPBA were found to be higher than CPPA [28]. In this work we report the effect of the anion size and their simultaneous controlled release property using zinc layered hydroxide as the host material, which can be employed as a new promising host delivery system for controlled release purpose similar to LDHs.

## 2. Experimental Section

### 2.1. Synthesis of ZLH Nanohybrid

All solutions were prepared using deionized water. The ZLH nanohybrid was prepared by the direct reaction of ZnO with the guest anions in an aqueous environment. About 1.00 g of pure commercial ZnO was reacted with 100 mL deionized water and mixed with 50 mL aqueous solution of 0.1 M DPBA and 0.1 M CPPA. The solution mixture of ZnO and the anions were stirred for 2 h before aging for 18 h at 70 °C in an oil bath shaker. The slurry was centrifuged, washed with deionized water, dried in an oven for 24 h and kept in a sample bottle for further use and characterization.

### 2.2. Herbicides Release Study

Simultaneous release of CPPA and DPBA from the ZCDX nanohybrid was studied by adding a 0.4 mg sample into 3.5 mL of 0.001–0.004 mol/L sodium carbonate aqueous solution. The quantity of phenoxyherbicides released into the solution was measured at the preset time at λ_max_ = 220.0 nm and 230.0 nm for CPPA and DPBA, respectively.

### 2.3. Characterization

Powder XRD patterns were recorded at room temperature with a Shimadzu XRD-6000 using filtered CuK_α_ radiation (λ = 1.5405 Å) at 40 kV and 30 mA, 2°·min^−1^. The FTIR spectra were collected in a Perkin-Elmer 1752X spectrophotometer using the KBr disc method, in the range 400–4000 cm^−1^. Thermal analyses (TGA-DTG) were performed using a Mettler Toledo instrument at a heating rate of 10 °C·min^−1^ between 35 °C and 1000 °C, under nitrogen flow of about 50 mL·min^−1^. A field emission scanning electron microscope (FESEM), Carl Zeiss Supra 40VP model was used to study the surface morphology of the materials.

**Figure 1:**
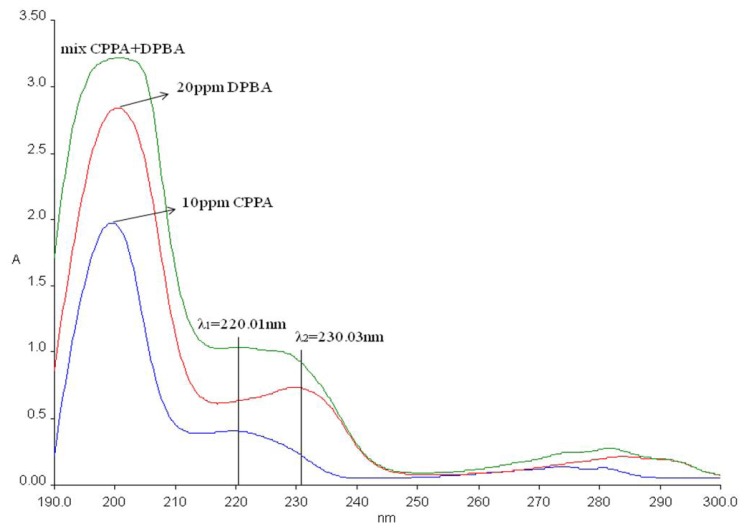
UV-vis spectra of pure anion for 2-(3-chlorophenoxy) propionate (CPPA) and 4-(2,4-dichlorophenoxy) butyrate (DPBA) at 220 and 230 nm, respectively.

The percentage loading of the dual herbicides intercalated into the ZCDX nanohybrid was determined using a Perkin Elmer UV-Visible Spectrophotometer, Lambda 35 at λ_max_ = 220.0 nm and 230.0 nm for CPPA and DPBA, respectively. The nanohybrid was dissolved into 2.0 M sodium carbonate aqueous solution so that both anions could exchange with CO_3_^2−^ and OH^−^ anions in the aqueous solution and release completely from the ZLH interlayers. Since the wavelengths of the pure herbicides is relatively close, there is an overlap between the two peaks (Figure 1), hence the data obtained was calculated by solving the simultaneous Equations 1 and 2,

(1) $$\text{at }\lambda_{1}:\;\;\;{\text{A}}_{1}={ɛ}_{\text{DPBA}}\times \text{b}\times {\text{C}}_{\text{DPBA}}+{ɛ}_{\text{CPPA}}\times \text{b}\times {\text{C}}_{\text{CPPA}}$$

(2) $$\text{at }\lambda_{2}:\;\;\;{\text{A}}_{2}={ɛ}_{\text{DPBA}}’\times \text{b}\times {\text{C}}_{\text{DPBA}}+{ɛ}_{\text{CPPA}}’\times \text{b}\times {\text{C}}_{\text{CPPA}}$$

where λ_1_ and λ_2_ are the wavelengths for DPBA and CPPA, respectively; A_1_ and A_2_ indicate the absorbance for the 100 ppm solution containing DPBA and CPPA at λ = 230 nm and λ = 220 nm, respectively, ɛ is the absorptivity of each anion, and C (mg/L) represents the concentration of 100% release of anions and b is the path length (1 cm). As shown in Equations (1) and (2), the concentration of DPBA and CPPA were obtained by solving the simultaneous equations, using ɛ_DPBA_ at λ_1_ and ɛ_DPBA_’ at λ_2_.

## 3. Results and Discussion

### 3.1. PXRD Analysis

**Figure 2:**
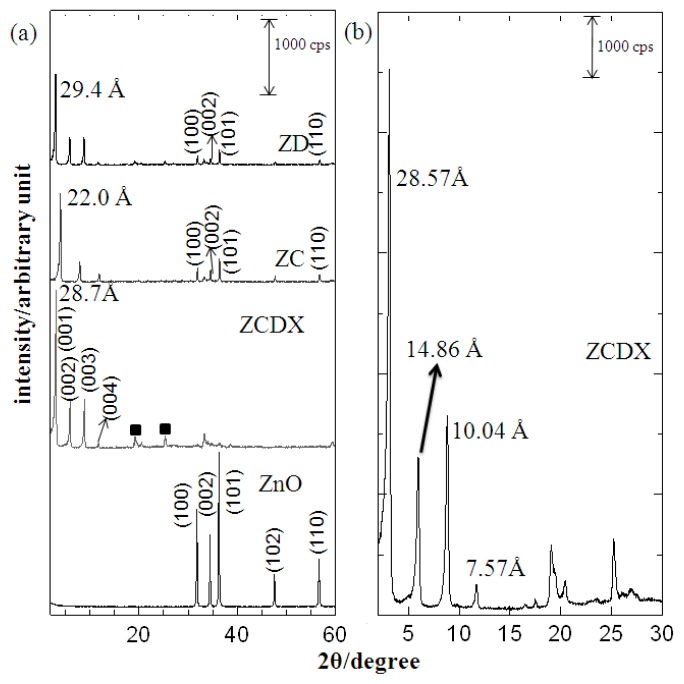
PXRD patterns of pure ZnO, a dual-guest nanohybrid ZCDX prepared at 0.1 M concentration of CPPA and DPBA anion, nanohybrid intercalated with single anion, CPPA (ZC) and DPBA (ZD) at 0.4 M CPPA and 0.2 M DPBA, respectively (**a**) together with slow scan of ZCDX (**b**). (■ refers to unknown phase).

The PXRD spectra and basal spacing for the ZLH nanohybrid intercalated with dual herbicides, CPPA and DPBA, labeled ZCDX together with single intercalation of both anions separately via direct reaction with ZnO are shown in Figure 2a.

Several studies has reported on the preparation of LHS by the hydrolysis of divalent metal salts and a metal oxide such as ZnO, followed by the inclusion of guest molecules into the host interlayers [6,7,12]. In our work, a one-step and direct reaction method, which involved dissociation*-*deposition mechanism, was adopted for the formation of the ZCDX nanohybrid [27,29]. ZnO was first added into the solution containing both anions. As a result it was hydrolyzed and formed Zn(OH)_2_ on the surface. The dissociation of Zn(OH)_2_ in the aqueous environment resulted in the release of Zn^2+^ and OH^−^. These two species then reacted with CPPA, DPBA and H_2_O in the solution to produce the layered ZLH-CPPA-DPBA nanohybrid compound.

Figure 2a shows very sharp and symmetric peaks for ZCDX at the lower 2θ angle due to the intercalation of CPPA and DPBA into the interlayer region. The PXRD patterns of ZCDX prepared at 0.1 M concentration of both anions display a high intensity diffraction peak indicating a pure phase material without any ZnO phase, especially the 100, 002 and 101 reflections [6,10,27]. This shows that a well-ordered nano-layered structure with good crystallinity was obtained at this optimum condition. A slow scan of ZCDX exhibits 4 harmonics at 2θ = 28.57 Å, 14.86 Å, 10.04 Å and 7.57 Å (Figure 2b), resulting in an average basal spacing of 29.67 Å. The obtained basal spacing value is higher than those reported for the intercalation of other type of herbicides into the LDH interlayers [20,26]. ZLH reportedly has larger interspacing than LDH to accommodate a greater number of incoming guest anions of varying sizes, due to its higher charge density [4,14,15]. Thus it is possible to simultaneously intercalate CPPA and DPBA anions into the ZLH interlayers.

### 3.2. Surface Morphology

**Figure 3:**
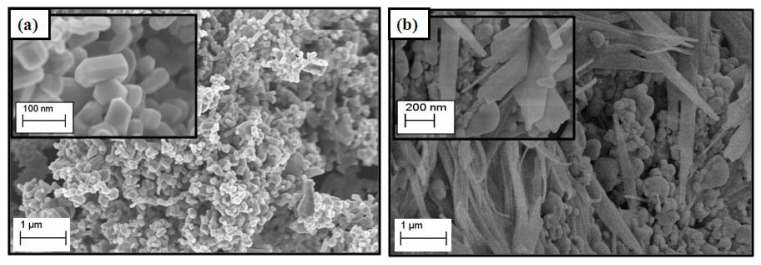
FESEM micrographs of ZnO (**a**) and zinc layered hydroxide (ZLH) nanohybrid (**b**) synthesized by direct reaction of ZnO at 10,000× magnification. The higher magnification of ZnO and ZLH nanohybrid are given in the insets.

The LHS can be described as morphologically having a plate-like structure, as reported elsewhere [10–12,15]. Figure 3 shows the surface morphology of pure commercial ZnO, exhibiting non-uniform granular structure with a particle size around 2 μm [27]. On simultaneous intercalation of the CPPA and DPBA anions into the ZLH interlayers, the ZnO phase changes in structure and was transformed into a mixture of granular and fiber-like structure. This shows that the transformation of ZnO into a nanohybrid produces surface morphology transformation.

### 3.3. Spatial Orientation of the Dual Herbicides in the ZLH Interlayers

**Figure 4:**
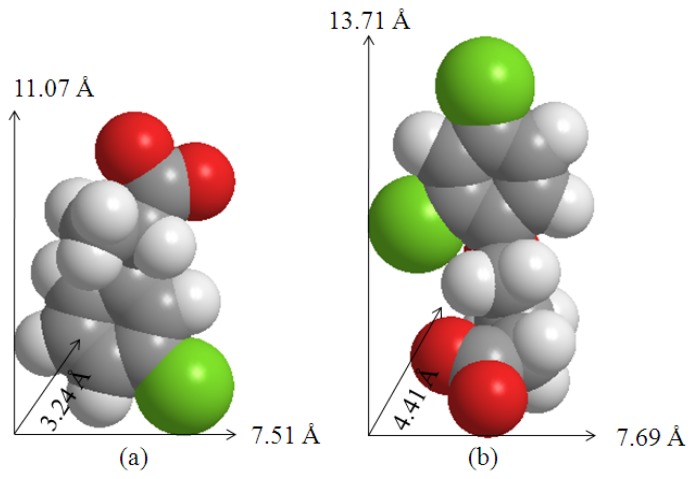
3D-Molecular structures of (**a**) 2-(3-chlorophenoxy)propionate; (CPPA) and (**b**) 4-(2,4-dichlorophenoxy)butyrate (DPBA).

**Figure 5:**
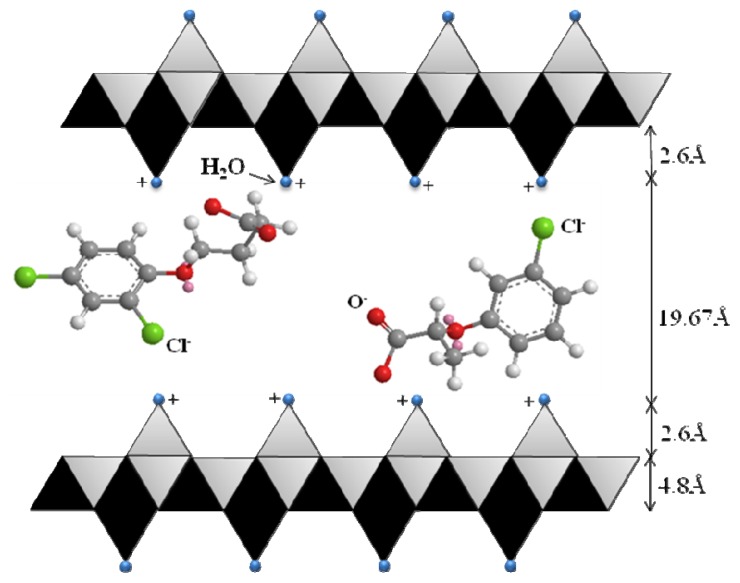
Proposed spatial orientation of CPPA and DPBA in the ZLH inorganic interlayers.

Figure 5 shows the proposed arrangement of the dual herbicides, CPPA and DPBA within the ZLH interlayer region. This is based on the PXRD data and the molecular size of both anions calculated using Chem3d Ultra 9.0 software [28] as shown in Figure 4. The ZLH is composed of inorganic layers with octahedral coordinated zinc cations, of which 1/4 are displaced out of the layer, leaving an empty octahedral site. Tetrahedrally coordinated Zn^2+^ located at the top and bottom of the octahedral sheet [4,5,7,11,17] are noted. Based on this structure, the inorganic layer thickness is 4.8 Å, including 2.6 Å for each zinc tetrahedron [9] and the basal spacing of the ZCDX nanohybrid is 29.67 Å. Therefore, the expected gallery height occupied by the two herbicides in the interlayer space of ZLH is 19.67 Å.

Considering several factors such as charge density of the layer, anion dimension and assuming that the layer structure remains intact after the intercalation of both anions [9,15], then the CPPA and DPBA have to orient themselves in a monolayer arrangement similar to those reported for salicylic acid with an interlayer spacing of 16.0 Å [14]. The oxygen atom in the carboxylate groups and also chlorine atoms attached to the benzene ring are directly bonded to the ZLH layers through hydrogen bonding and electrostatic interaction. Such an arrangement leads to stronger interaction between the host layers and both the anions which cause the increase in thermal stability of the layered structure [3,11,14,24].

### 3.4. Thermal Studies

**Figure 6:**
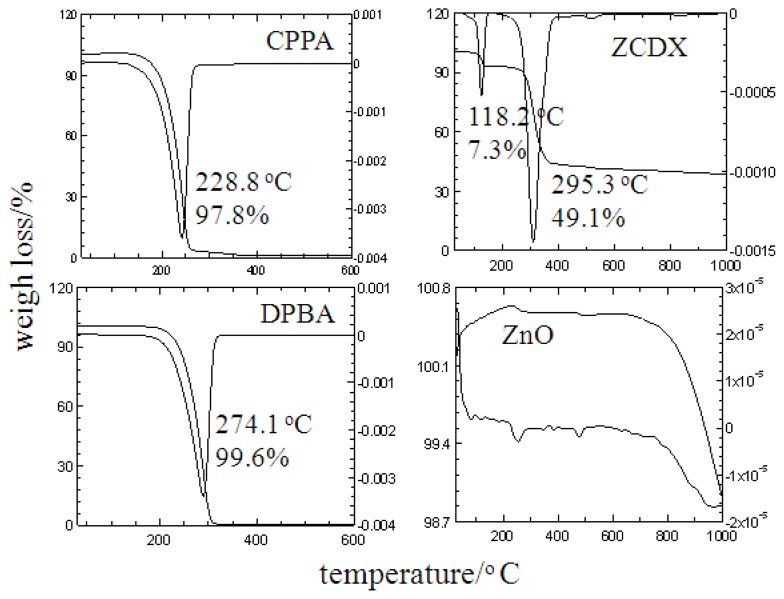
TGA-DTG thermograms of CPPA, DPBA, dual-guest nanohybrid ZCDX and ZnO.

The thermal decomposition behavior of ZnO and ZCDX nanohybrid was investigated using TGA-DTG studies. It is known that the LHS thermograms are generally described by two thermal events which are similar to that for organo-LDHs [5,12]. The first endothermic peak is observed with the temperature range of room temperature to nearly 200 °C, commonly assigned to the loss of adsorbed and structural water molecules of the ZLH layers. At approximately 300 °C, the weight loss is attributed to simultaneous decomposition of anions and dehydroxylation of the zinc hydroxide layer [1,4,8,9]. As shown in Figure 6, no thermal decomposition was observed for pure ZnO, which proved that it is a thermally stable compound. CPPA and DPBA anions show an endothermic peak at temperature maxima of 228.8 °C and 274.1 °C with weight loss of 97.8% and 99.6%, respectively.

The TG curve of ZCDX shows two major weight losses at temperature maxima of 118.2 °C and 295.3 °C. The first stage of weight loss with 7.3% is ascribed to the removal of the surface and intercalated structure of water molecules, leading to the decomposition for dual herbicides of the nanohybrid at 295.3 °C with 49.1% weight loss. This shows that the ZCDX nanohybrid is thermally more stable than CPPA and DPBA in their salt form [9,26]. DPBA has higher thermal stability since it decomposed at a higher temperature compared to the CPPA anion. We assumed that the DPBA anion has higher stability and stronger interaction with the ZLH interlayer compared with the CPPA due to the two chlorine atoms attached to the benzene ring, which may induce stronger polar interaction with ZLH layers based on the proposed orientations of both anions in the interlayers as mentioned earlier.

### 3.5. FTIR Analysis

**Figure 7:**
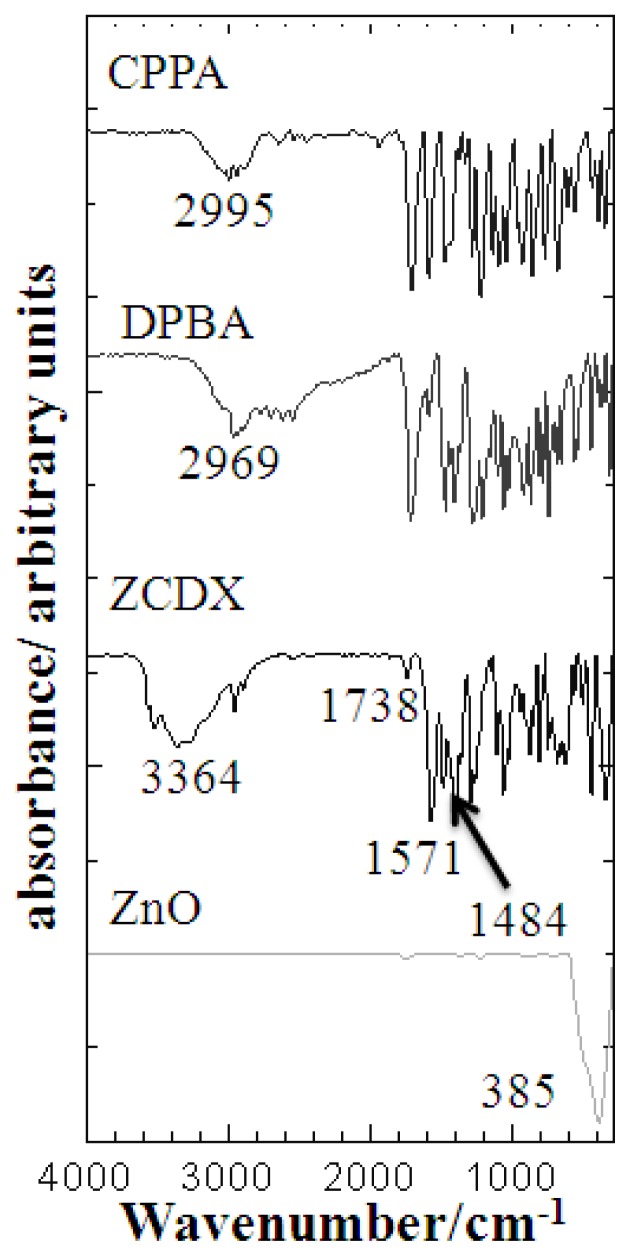
FTIR spectra of the guest anions CPPA, DPBA, the nanohybrid ZCDX and ZnO.

The presence of the two herbicides in the ZLH interlayers was also elucidated by FTIR spectroscopy, which supported the PXRD results [2]. Figure 7 compares the FTIR spectrum of pure ZnO, intercalated compound, ZCDX with CPPA and DPBA anions. The sharp and intense peak of ZnO at the lower wavenumber range, 385 cm^−1^ is due to the Zn–O sublattice stretching vibration [8,27]. Similar absorption bands could also be observed for the CPPA and DPBA anions, as they possess the same functional groups. Broad peaks at 2995 and 2969 cm^−1^ are attributed to the OH stretching vibration of COOH for CPPA and DPBA anions, respectively. Intense peaks at around 1700 cm^−1^, for CPPA (1705 cm^−1^) and DPBA (1714 cm^−1^) are ascribed to C=O stretching vibration of the protonated COOH group of herbicides. Strong bands at 1471 and 1467 cm^−1^ are due to the stretching vibration of the aromatic ring, C=C and bands at around 1200 cm^−1^ are due to C–O–C antisymmetric and symmetric stretching.

As expected, ZCDX has an absorption spectrum similar to the herbicide anions. However, some bands are slightly shifted due to the interaction of both the anions and host layers. Weak bands at around 1700 and 1400 cm^−1^ are due to the stretching vibration of the C=O of the COOH group and C=C stretching of the aromatic ring, as mentioned earlier, indicating that the two herbicides were successfully intercalated into the ZLH interlayers [20]. A strong peak at 1571 cm^−1^ is due to the antisymmetric and symmetric carboxylate stretching of the anion. At the lower wavenumber, the disappearance of the broad ZnO peak in the nanohybrid confirmed that all the ZnO had reacted with the CPPA and DPBA, resulting in the formation of the nanohybrid, which concurs with the PXRD data.

### 3.6. Herbicides Release Properties

**Figure 8:**
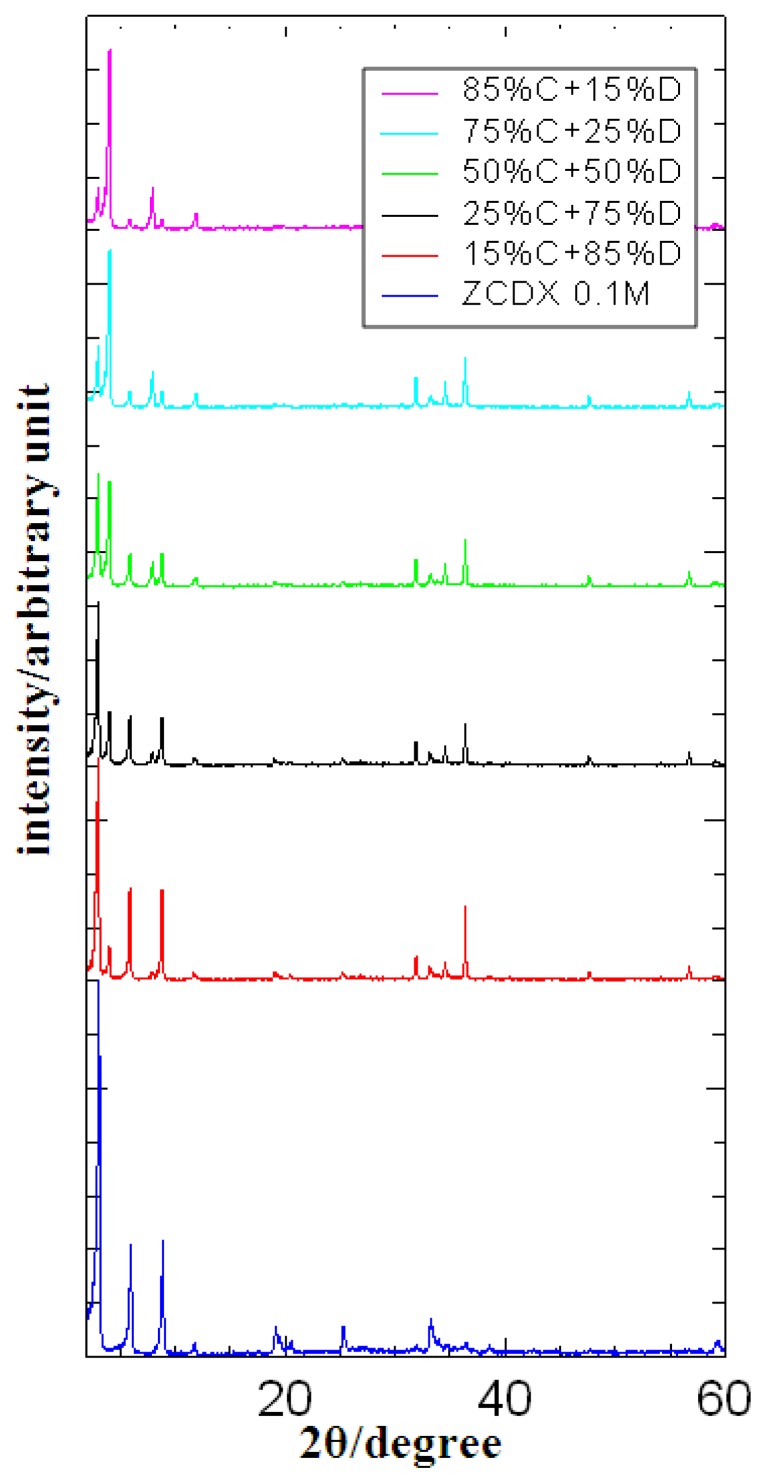
PXRD patterns of ZCDX nanohybrid and simulated by addition of PXRD patterns of single intercalation nanohybrid ZC and ZD at different percentages.

The loading percentages of CPPA and DPBA were analyzed using a UV-Vis spectrophotometer and the data were calculated using simultaneous equations, as stated earlier. The data show that the ZCDX nanohybrid is composed of CPPA and DPBA with percentage contribution of 16.22% and 83.78%, respectively. The percentage represents the ratio of CPPA and DPBA intercalated into ZLH. The simulation data of both single intercalations of ZC and ZD nanohybrids at different percentages (Figure 8) shows that a good fit was obtained when the PXRD patterns were obtained using 15% CPPA and 85% DPBA contribution. The high content of the DPBA anion reflects the higher abundance of DPBA in the interlayer gallery space of ZLH compared with the CPPA and this in turn will influence the release behavior.

**Figure 9:**
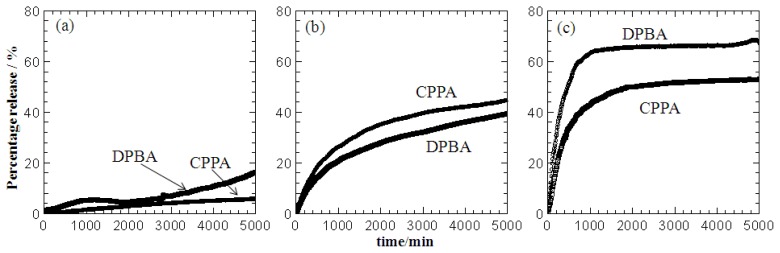
Release profiles of CPPA and DPBA from the dual-guest nanohybrid, ZCDX into various concentrations of Na_2_CO_3_ aqueous solution: 0.001 mol/L (**a**), 0.002 mol/L (**b**), and 0.004 mol/L (**c**).

The simultaneous release of CPPA and DPBA from the nanohybrid lamellae into the release media was performed in various concentrations (0.001–0.004 mol/L) of sodium carbonate aqueous solution. The affinity of CO_3_^2−^ and OH^−^ anions in the aqueous solution is higher than the CPPA and DPBA towards the ZLH interlayers; therefore, both herbicides anions can be ion-exchanged with the carbonate [2,20,22,25] and the release of CPPA and DPBA was determined at λ_max_ = 220 and 230 nm, respectively. Generally, the release rate is initially rapid, and then slows until it reaches equilibrium. The percentage release of CPPA and DPBA increased when the ZCDX was placed in a higher concentration of sodium carbonate, as shown in Figure 9a–c. Maximum release was achieved at about 2000 min (approximately after a day) into 0.004 mol/L sodium carbonate. Taking the intercalated amount of the CPPA and DPBA in ZCDX as 100% respectively, the maximum accumulated release of CPPA was found to be 53.3% which is less than DPBA with maximum saturated release of 68%.

Previous work revealed that release behaviors of different drugs from the same host are quite different [24]. Such behavior could be related to several factors such as the guest size, preparation method of the nanohybrid, anion affinity in the release medium, particle size, packing density and chemical interactions between the host and guest [15,17,24–26]. Our results clearly proved that the release of the CPPA and DPBA depends on the anionic size as well as on the host-guest and/or guest-guest interaction between the host interlayers. The high uptake of DPBA anion is due to its larger size and stronger interaction within the high-charged density of the ZLH interlayers when two Cl^−^ atoms bonded with Zn^2+^ ions compared to only one Cl^−^ atom in the CPPA [28], as proposed in Figure 5. The abundance of DPBA anion (high loading percentage) makes it easier to be released in high concentration sodium carbonate solution.

**Figure 10:**
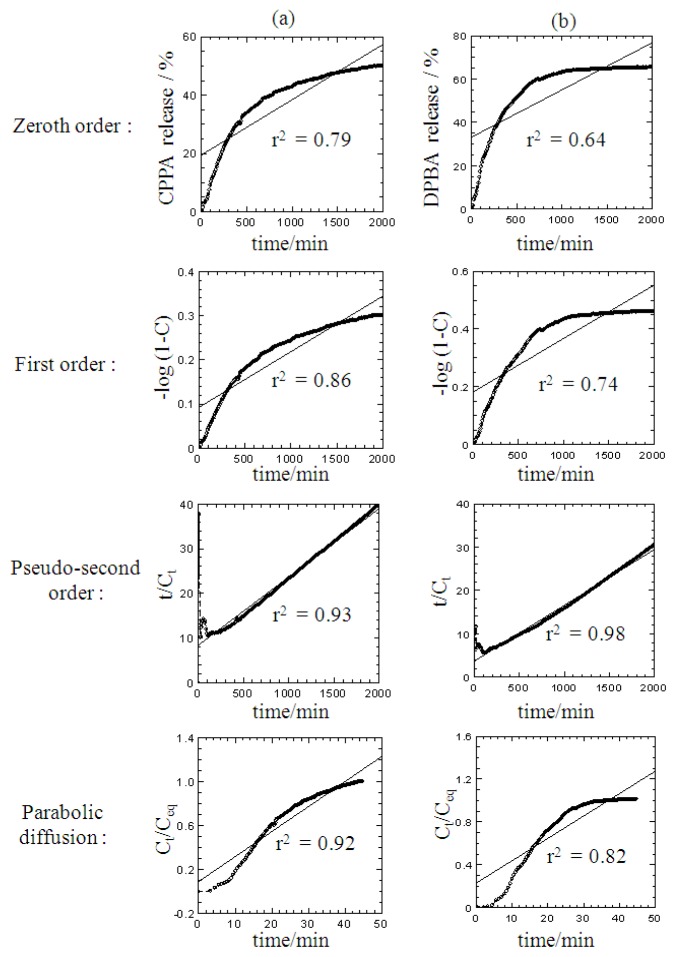
Fitting the release data of CPPA (**a**) and DPBA (**b**) from ZCDX nanohybrid into 0.004 mol/L Na_2_CO_3_ solution to the zero-, first-, pseudo second-order kinetics and parabolic diffusion model.

The data of the cumulative release of the guests were fitted to several kinetic models and plotted in Figure 10. Among the models used in this work, the correlation coefficient (*r*^2^) for the pseudo second-order kinetic gave the best fit for both CPPA and DPBA, as shown in Table 1. The half-life, *t*_1/2_ values for the DPBA and CPPA which are 286 and 543 min, respectively, confirmed that the former is easier to release than the latter from the nanohybrid, when both anions are present simultaneously. In most of controlled release formulation, a large amount of anions (in this case, DPBA and CPPA) were released at the very beginning of the release time before the release rate reaches a stable profile. This is due to the swelling of the porous structure followed by the dissolution of the anions. This phenomenon is referred to as “burst release” [23], which happens in a very short period of time compared to the entire release process, resulting in the deviation from linearity of the pseudo-second order fit at the beginning of the release process.

## 4. Conclusions

In conclusion, the monophasic, well-ordered ZCDX nanohybrid containing two herbicides, CPPA and DPBA, was found to be composed of a higher loading of DPBA compared to CPPA between the ZLH inorganic interlayers with percentage contribution of 83.78% and 16.22%, respectively. Simultaneous release of CPPA and DPBA from ZCDX into sodium carbonate aqueous solution was found to be controlled by pseudo second-order kinetics. The release rate of both CPPA and DPBA was found to be different, suggesting that the anionic guest molecules sizes and the interactions between the host and guest could control the release kinetics. The present results demonstrate the potential application of a layered single metal hydroxide, particularly zinc layered hydroxide as the host for the preparation of a nanohybrid compound with tunable controlled release property containing two herbicides simultaneously.

## Figures and Tables

**Table 1:** Correlation coefficient, *r*^2^ values obtained from fitting the data of simultaneous release of CPPA and DPBA to zero-, first- and pseudo-second order kinetics. The half-life, *t*_1/2_, k and c values for pseudo-second order kinetics are also shown.

Models	Zeroth Order	First Order	Parabolic Diffusion	Pseudo-Second Order			
Anion	*r*^2^			*r*^2^	*t*_1/2_ (0−3000 min)	k (×10^−5^) (Lmg^−1^min^−1^)	c

CPPA	0.79	0.86	0.92	0.93	543	2.82	8.31
DPBA	0.64	0.74	0.82	0.98	286	4.51	3.69

equations							

Zeroth order :C=kt+c				$\text{Pseudo}-\text{second order}:\text{t}/{\text{C}}_{\text{t}}=1/{\text{k}}_{2}{{\text{C}}_{\text{eq}}}^{2}+(1/{\text{q}}_{\text{e}})\;\text{t}$			
First order:-log(1-C)=kt+c				$\text{Parabolic diffusion}:{\text{C}}_{\text{t}}/{\text{C}}_{\text{eq}}=\text{c}+{\text{kt}}^{0.5}$			

C_eq_ = concentration of anion at equilibrium, C_o_ = initial concentration of the anions, C_t_ = concentration of anion at time t, C = percentage release of anion, c = a constant.
